# Effects of Introducing Generative AI in Rehabilitation Clinical Documentation

**DOI:** 10.7759/cureus.81313

**Published:** 2025-03-27

**Authors:** Kyohei Omon, Tomohiko Sasaki, Ryota Koshiro, Takeshi Fuchigami, Masahiro Hamashima

**Affiliations:** 1 SDX Research Institute, Seiwakai Medical Corporation Group, Osaka, JPN; 2 Department of Rehabilitation, Saito Rehabilitation Hospital, Osaka, JPN; 3 Department of Rehabilitation, Kawanishi Rehabilitation Hospital, Hyogo, JPN; 4 Department of Rehabilitation, Kishiwada Rehabilitation Hospital, Osaka, JPN

**Keywords:** clinical documentation, generative artificial intelligence, healthcare professionals, operational efficiency, rehabilitation, workload

## Abstract

Introduction

Healthcare professionals reportedly spend a significant proportion of their working hours on documentation. Therefore, we developed a generative AI solution specialized in creating clinical documentation for rehabilitation. This study aimed to examine the impact of generative AI on clinical documentation tasks.

Methods

Twelve rehabilitation professionals (physical therapists, occupational therapists, and speech-language pathologists) participated in this study. We compared conventional clinical documentation (Period A) with clinical documentation using a generative AI system (Period B). Measures taken for both periods included time required to complete the clinical documentation (documentation time), workload assessed using the National Aeronautics and Space Administration Task Load Index (NASA-TLX), and quality of the clinical documentation. Between-group comparisons of these measurements were performed. Additionally, we recorded the number of non-conversational voice memos (voice data inputs) in Period B. After the study, we assessed the participants’ willingness to adopt generative AI (implementation intent) on a five-point scale. For statistical analysis, we compared documentation time, NASA-TLX scores, and documentation quality between the two periods. Time saved was determined by subtracting the documentation time of Period B from that of Period A, and a correlation analysis between the number of voice memos (voice data input) and the willingness to adopt the technology was conducted. Analyses were performed using R version 4.2.3 (R Core Team, Durham, NC), with the level of significance set at 0.05.

Results

No significant difference was observed in the time required to prepare clinical documentation between Periods A and B. However, in Period B, the NASA-TLX time pressure score was significantly lower, while the quality of clinical documentation was significantly higher. Additionally, a strong positive correlation was observed between the reduction in documentation time and the number of voice memos (r = 0.71, p < 0.01), as well as a significant positive correlation with the willingness to adopt the system (r = 0.67, p < 0.05) during clinical documentation in Period B.

Conclusion

Our findings indicate that using generative AI for clinical documentation tasks can reduce time pressure and improve documentation quality. Moreover, the reduction in documentation time was associated with the frequency of voice memos and the degree of participants’ willingness to adopt the system. These results suggest that, to achieve further reductions in workload and costs, considering the motivation and cooperative framework of healthcare professionals when introducing generative AI solutions is essential.

## Introduction

In medicine and rehabilitation, reducing workload and improving efficiency have been gaining rapid importance. It has been reported that healthcare professionals spend as much as 25%-40% of their working hours on documentation, and by reducing the time required for this task, they can devote more time to patient care, potentially improving the quality of that care [1,2]. This demand continues to grow alongside the rapid advancement of medical technology, arising from the wide range of tasks Healthcare professionals must undertake, including diagnosis, treatment, and care planning.

Recently, large language models (LLMs) that employ natural language processing have drawn attention as a potential solution for reducing workload in the medical field [3]. LLMs, a type of deep learning model used in natural language processing, can generate and comprehend natural language by learning from vast amounts of textual data. These LLMs have also demonstrated certain achievements in generating specialized medical terminology [4].

However, constructing LLMs requires an enormous amount of text data, making it challenging to apply existing generative AI tools in the rehabilitation domain, where specialized terms and domain-specific language are prevalent. Consequently, most studies on clinical documentation using existing generative AI have been conducted outside the rehabilitation field, and research focusing on rehabilitation professionals is limited.

This study aimed to evaluate whether the use of generative AI reduces clinical documentation time, alleviates workload, and improves quality in a rehabilitation setting. To achieve this, we utilized “medimo” (Pleap Inc., Tokyo, https://pleap.jp/), a generative AI system leveraging LLMs that have already been implemented in the medical domain. This innovative endeavor has the potential to enhance the quality of rehabilitation services and to alleviate the workload of healthcare professionals.

## Materials and methods

Participants

This prospective comparative study compared a conventional clinical documentation period with a generative AI-assisted documentation period. The participants consisted of 12 healthcare professionals, including two physical therapists, two occupational therapists, and two speech-language-hearing therapists from Saito Rehabilitation Hospital and Kawanishi Rehabilitation Hospital. To ensure familiarity with the work environment and a sufficient understanding of their professional duties, only those who had been employed at their respective institutions for at least six months and had a minimum of five years of clinical experience were included. The average participant age was 33.2 ± 6.8 years, with an average of 10.1 ± 3.1 years of professional experience; four were male and eight were female. Additionally, an evaluator, who was different from the main researcher, was assigned to each hospital.

Equipment

During the generative AI-assisted documentation period, we used “medimo” (developed by Pleap Inc.; https://pleap.jp/). Medimo is a web application that streamlines the clinical documentation process by transcribing voice inputs from healthcare professionals during patient consultations and summarizing the content using AI. In collaboration with Pleap Inc., we enhanced the existing version of medimo by training it with a newly collected speech dataset specifically designed for rehabilitation professionals. Evaluation confirmed that the accuracy of documents generated by the enhanced medimo was at least equivalent to or greater than that of the original version.

Procedure

**Figure 1:**
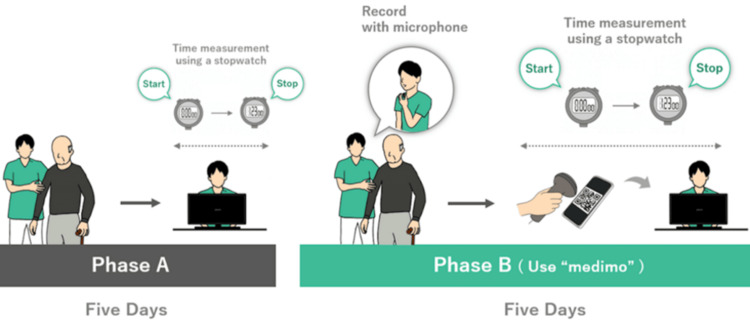
Procedure using an AB design During Phase A, participants created and registered clinical documentation in their usual manner, and the time required to complete and register the documentation was measured. In Phase B, voice data from the consultation was recorded, and clinical documentation was created using medimo. The time was measured from scanning the QR code output by medimo to transcribing it into the clinical documentation and registering the record. Image credit: Kyohei Omon. Permission granted for use in this article.

The study was conducted using an AB design (Figure 1). First, the participants performed clinical documentation as usual for five days (Phase A). During this period, participants created and registered clinical documentation in the usual manner. After completing Phase A, an investigator familiar with medimo provided instructions to each participant regarding its use. The procedure for using medimo was as follows: (1) attach a speech-recording microphone, (2) launch the medimo application, (3) conduct the usual consultation, (4) record additional information to be included in the clinical documentation, (5) operate the medimo app to summarize the recorded content, (6) display the summary as a QR code, (7) scan the QR code using an electronic medical record (EMR) system, and (8) edit and register the summary in the EMR. Participants underwent training for one to three days to become proficient in these procedures. Afterward, in Phase B, participants recorded voice data during consultations and used medimo to create clinical documentation.

Outcome

The evaluation items were (1) clinical documentation creation time, (2) workload as measured by the National Aeronautics and Space Administration Task Load Index (NASA-TLX), and (3) the quality of the clinical documentation.

The input time was measured by a third party, starting when the participant sat at a personal computer with an electronic medical record system ready for documentation and ending when the registration of the clinical documentation was complete. Participants were instructed to create the clinical documentation as accurately as possible and avoid performing tasks other than documentation. The input time was measured daily, and the average time for each phase was calculated.

The NASA-TLX, developed by Hart and Staveland [5], is a subjective assessment method comprising six subscales: Mental Demand (MD), Physical Demand (PD), Temporal Demand (TD), Own Performance (OP), Effort (EF), and Frustration Level (FR) [6]. Each item was rated on a 0-100 Visual Analog Scale (VAS). For the VAS, a single line segment with 20 tick marks (in increments of 5) was labeled from “0: low” to “100: high,” and participants were asked to place a mark anywhere along the line. If a mark appeared between two tick marks, the value was rounded to a higher number, and that value was recorded for each item. The NASA-TLX was measured daily, and the average scores for each phase were calculated.

**Figure 2:**
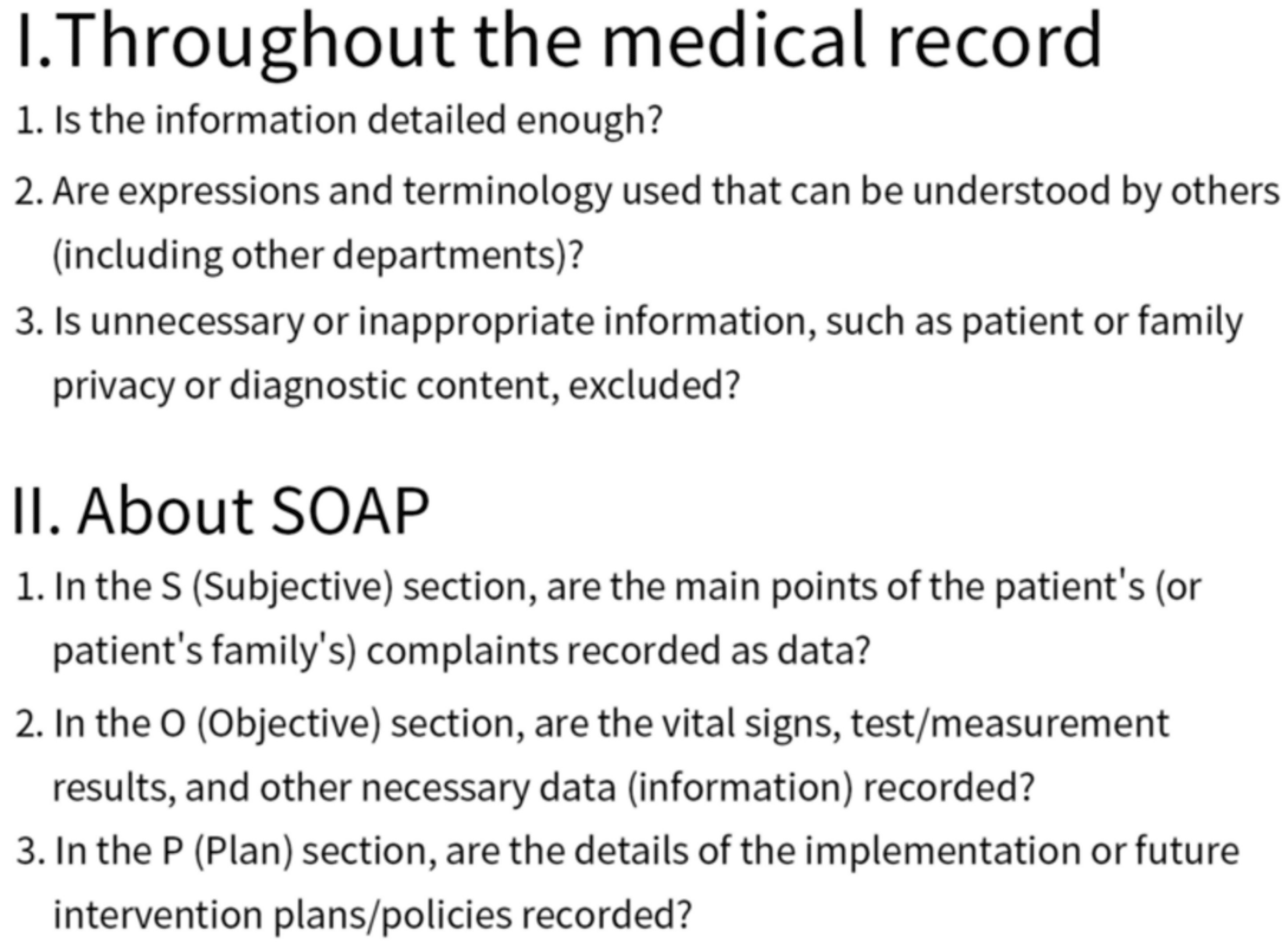
Evaluation scale used for assessing the quality of clinical documentation Three physical therapists who were not involved in the study participated in the scoring. The evaluated clinical documentation was created for each participant during the study period, and the creation time was measured. Six question items were scored on a 10-point scale, and the average score was calculated based on ratings from the three scorers.

The clinical documentation quality was assessed based on the records created during the input time measurement period, focusing on the level of detail in the progress notes, appropriateness of expressions and terminology, privacy considerations, and adherence to the SOAP format. Three physical therapists not involved in this study assigned scores using a six-item rating sheet created by our team, with each item rated on a 10-point scale (Figure 2). The average score of each participant in each phase was calculated. Notably, the “Assessment (A)” component of SOAP was excluded from this study. The reasons were: (1) the method of documenting “A” varies both among and within hospitals, and (2) it involves significant professional discretion, making objective assessment difficult. Because of these factors, it was challenging to evaluate “A” using a common objective standard; therefore, only S, O, and P were evaluated in this study.

After the study was concluded, we determined the number of non-conversational audio memos (audio data input) recorded by each participant during Phase B and conducted a survey on the participants’ willingness to adopt medimo. Willingness was gauged by asking, “If medimo were introduced, would you use it?” with the following options: “Definitely would use” (5 points), “Probably would use” (4 points), “Unsure” (3 points), “Probably would not use” (2 points), and “Definitely would not use” (1 point).

Statistical analysis

We used the average values for the input time, NASA-TLX, and quality of clinical documentation to perform between-group comparisons. Additionally, we calculated the reduction in documentation creation time by taking the difference between Phases A and B. Correlation analyses with both the frequency of audio memos (audio data input) and the willingness to adopt the system were also conducted. Each dataset was checked for normality and homogeneity of variance. Parametric tests were applied to parametric data, and nonparametric tests were applied to nonparametric data. Analyses were performed using R (version 4.2.3; R Core Team, Durham, NC), with statistical significance set at p < 0.05.

Ethical considerations

This study was approved by the Ethics Committees of the Saito Rehabilitation Hospital and Kawanishi Rehabilitation Hospital (approval numbers: 2024-1 for Saito Rehabilitation Hospital, 2023-1 for Kawanishi Rehabilitation Hospital). All participants provided written informed consent.

## Results

**Figure 3:**
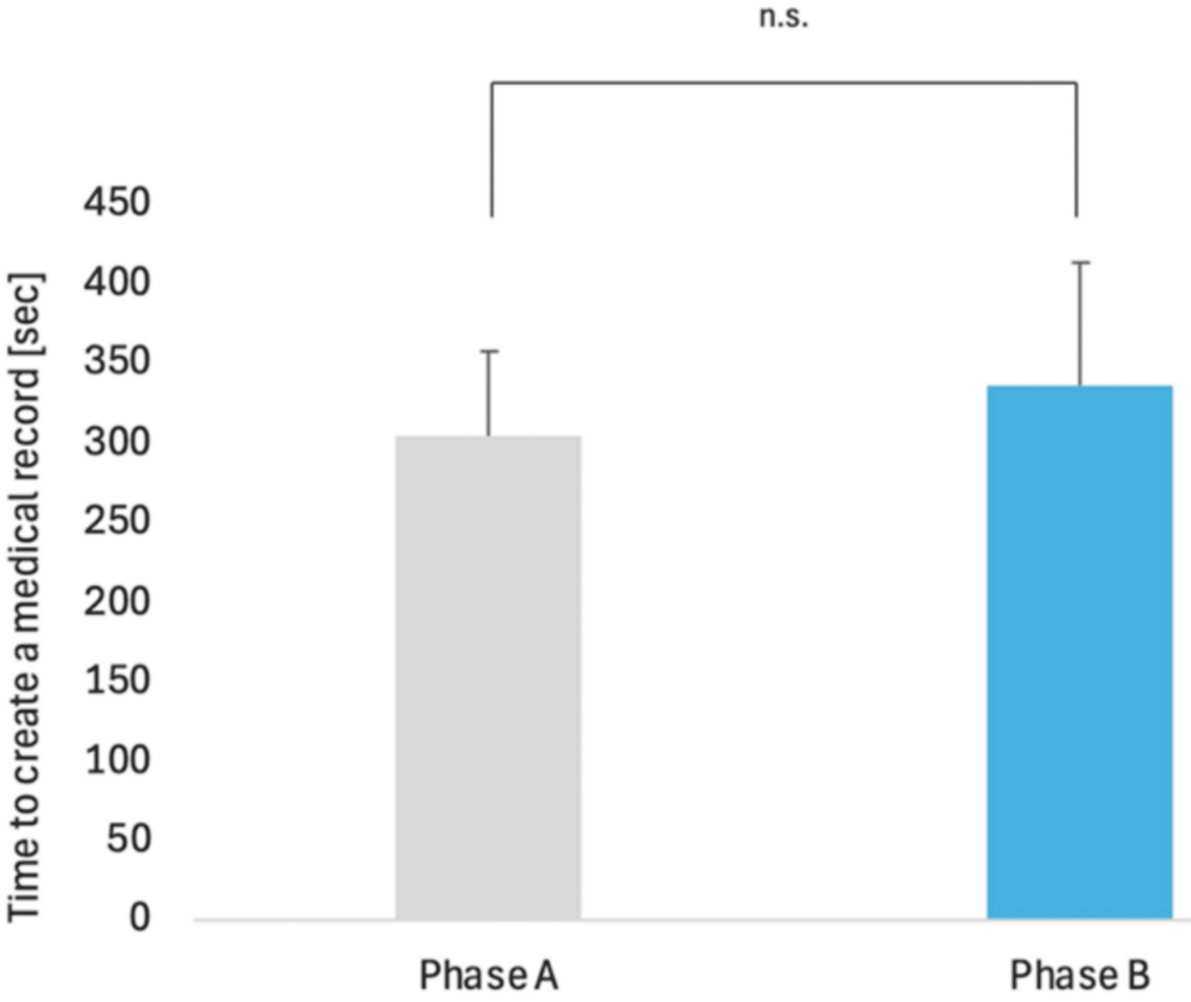
Clinical documentation time 305 s in Period A and 336 s in Period B, with no significant difference. n.s.: not significant

No adverse events were observed during clinical documentation using medimo. The documentation time was 305 ± 53.9 s in Period A and 336 ± 78.0 s in Period B, with no significant difference between the periods (Figure 3).

In the NASA-TLX assessment of workload, no significant differences were observed in mental demand, physical demand, task performance, or effort (Table 1). However, time pressure was significantly lower when medimo was used (p = 0.037), whereas dissatisfaction was significantly higher (p = 0.041).

**Table 1:** Results of NASA-TLX Abbreviations: MD, mental demand; PD, physical demand; TD, temporal demand; OP, own performance; EF, effort; FR, frustration level; AVE, average; SD, standard deviation. *Significant difference between phases A and B.

Participant	MD		PD		TD *		OP		EF		FR *	
ID	Phase A	Phase B	Phase A	Phase B	Phase A	Phase B	Phase A	Phase B	Phase A	Phase B	Phase A	Phase B
1	73	48	73	44	77	44	28	46	73	47	64	50
2	70	67	50	69	73	60	60	58	65	69	68	63
3	38	61	21	50	34	55	40	57	33	60	29	58
4	39	48	39	49	46	51	45	51	44	49	37	46
5	65	59	55	58	69	59	60	56	72	68	62	67
6	49	30	43	28	38	30	27	29	41	34	23	42
7	46	60	48	45	84	80	32	32	44	48	40	52
8	53	58	51	39	49	39	31	43	64	56	54	61
9	56	51	10	14	55	29	41	42	67	57	41	55
10	51	50	47	48	61	55	39	48	49	54	49	62
11	79	70	73	70	85	77	80	76	85	74	85	80
12	20	22	5	13	25	19	15	29	36	28	5	27
AVE	53.3	52.0	42.9	43.9	58.0	49.8	41.5	47.3	56.1	53.7	46.4	55.3
SD	16.1	13.5	20.7	17.6	19.2	17.8	17.0	13.1	16.2	13.1	21.0	12.9

Regarding the quality of clinical documentation, the scores for SOAP sections S and P, as well as the total score, were significantly higher when medimo was used (p = 0.045, p = 0.045, p = 0.023; Table 2).

**Table 2:** Results of clinical documentation quality Abbreviations: SOAP_S, subjective of SOAP; SOAP_O, objective of SOAP; SOAP_P, plan of SOAP; AVE, average; SD, standard deviation. *Significant difference between phases A and B.

Participant	Details		Non use technical jargon		Appropriate expressions		SOAP_S *		SOAP_O		SOAP_P *		Total *	
ID	Phase A	Phase B	Phase A	Phase B	Phase A	Phase B	Phase A	Phase B	Phase A	Phase B	Phase A	Phase B	Phase A	Phase B
1	7.0	6.7	7.0	5.7	8.3	8.7	2.0	7	7.7	7	2.7	6	34.7	41.1
2	7.7	7.3	8.7	7.7	8.7	8.3	2.3	8.3	8.3	7.3	3.0	7	38.7	45.9
3	4.7	7	7.0	8	7.3	8.3	2.0	5.7	6.0	7	2.7	6	29.7	42
4	7.0	8.3	6.7	8.7	8.3	8.7	3.0	8	6.7	7.3	2.3	7.3	34.0	48.3
5	8.0	8	7.0	7	8.3	8.3	2.3	5.7	8.3	7.7	2.7	3	36.6	39.7
6	7.0	7.7	7.0	7.3	8.3	8.3	2.3	2	7.3	8	2.7	2.7	34.6	36
7	7.7	7	7.7	7.7	8.3	8.3	9.3	8	9.0	8.7	7.7	7.3	49.7	47
8	6.7	8.3	8.0	8	8.3	8.7	8.0	9	7.0	7.7	8.7	9.3	46.7	51
9	7.0	6.3	8.0	7	8.3	8.7	6.7	7.3	7.3	7	7.0	8	44.3	44.3
10	7.3	7.3	8.0	8	8.3	8	7.7	8.7	7.7	7.3	8.7	6.7	47.7	46
11	8.0	7.7	8.3	8.3	8.3	8.3	8.3	7.7	7.7	8	8.0	9	48.6	49
12	8.0	8.3	6.0	7.7	8.7	8.3	8.0	8	7.0	8.3	8.0	8.3	45.7	48.9
AVE	7.2	7.5	7.5	7.6	8.3	8.4	5.2	7.1	7.5	7.6	5.4	6.7	40.9	44.9
SD	0.9	0.6	0.7	0.7	0.3	0.2	2.9	1.8	0.8	0.5	2.7	2.0	6.6	4.3

**Figure 4:**
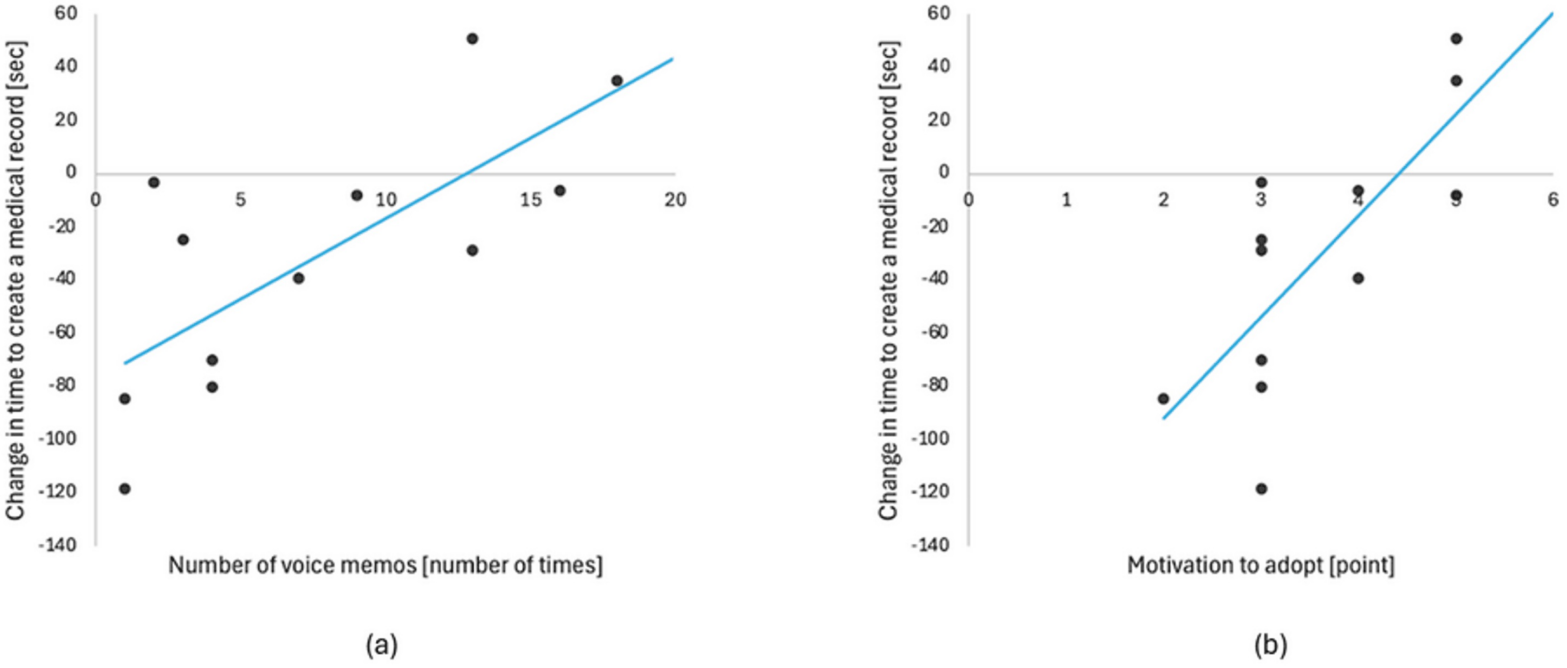
Relationship with the reduction in clinical documentation time (a) Correlation with the number of voice memos. A strong positive correlation was observed (r = 0.71, p = 0.006). (b) Correlation with the willingness to adopt the system. A significant positive correlation was observed (r = 0.67, p = 0.009).

Additionally, during clinical documentation with medimo in Period B, a strong positive correlation was observed between the reduction in documentation time and the number of voice memos (r = 0.71, p = 0.006; Figure 4a) and a significant positive correlation between the reduction in documentation time and the willingness to adopt the system (r = 0.67, p = 0.009; Figure 4b).

## Discussion

This study examined whether the use of generative AI affects the time, burden, and quality of clinical documentation in rehabilitation. The results showed that using medimo decreased time pressure and improved documentation quality. Additionally, the number of voice memos during medimo use and the willingness to adopt the system were found to influence the reduction in documentation time, suggesting that user skill and adoption motivation must be considered to achieve time savings.

Documentation time was 305 ± 53.9 s in Period A and 336 ± 78.0 s in Period B, with no significant difference between periods. AI technology implementation is expected to create an environment in which AI automatically organizes entered data, and when voice input is sufficient, the record creation process can be shortened [7]. However, in this study, insufficient voice input necessitated additions and corrections to the documentation summarized by medimo, and these editing tasks were time-consuming. Furthermore, educational and awareness activities aimed at improving literacy are considered urgent for effective AI utilization [8]. One reason why medimo usage did not lead to reduced documentation time in this study may be the insufficient acquisition of AI utilization skills among the staff. Specifically, staff may have experienced challenges verbalizing their thoughts when using medimo's voice memo function or were uncertain about when to record, potentially leading to less frequent utilization. This likely contributes to the inability to fully realize documentation efficiency gains.

When new systems are introduced in healthcare settings, productivity tends to decrease [9], with maximum benefits occurring only after the staff members gradually accept these systems [10]. Although this study found no significant difference in documentation time, a significant positive correlation was observed among willingness to adopt, the number of voice memos, and time reduction. These results suggest that documentation time could potentially be reduced when users are receptive to medimo and actively use it during interventions.

Time pressure was significantly lower when medimo was used. In Japanese rehabilitation specialty hospitals, clinical documentation is often completed between treatment sessions, with limited time between sessions, to maintain efficient service delivery. This likely contributes to perceived time pressure in documentation, which was scored higher than the other items in our results. Introducing medimo into the documentation process allows AI to summarize clinical records to some extent, thus eliminating the need to create records from scratch. This appears to have reduced the time pressure associated with documentation, suggesting potential contributions to reducing therapists' workloads.

Conversely, dissatisfaction was significantly higher when using medimo. For healthcare staff, the urgency of implementing ICT is not necessarily high, and their ICT literacy is low [11]. In this context, using the AI tool, medimo, which differs from conventional documentation methods, may have generated feelings of anxiety and stress due to insufficient skill mastery among the staff.

Documentation quality scores for SOAP sections S and P as well as the total score were significantly higher when using medimo. The subjective complaints of patients (S in the SOAP) and specific treatment content (P in the SOAP) should accurately reflect what occurs during treatment. Therefore, medimo, which allows real-time recording during treatment sessions, is likely to result in more accurate documentation.

This study had a few limitations. First, there were sample size constraints. This was a small-scale investigation with 12 participants, and caution is required when generalizing the results. Second, there were research design constraints. In this study, both Periods A and B had very short observation periods of 5 d, which may have been insufficient to evaluate long-term effects and participants' adaptation processes. Third, AI literacy and education are insufficient. The participants had limited time to thoroughly learn how to use medimo, and prior education for improving AI literacy was inadequate. Additionally, the method of evaluating documentation quality included subjectivity. The documentation evaluation criteria depended on the evaluators' subjective judgments, potentially causing variations in the evaluation results owing to different standards and interpretations among evaluators. Lastly, the reliability and validity of the evaluators were not rigorously examined, representing a limitation in subjective evaluation.

## Conclusions

The use of generative AI in clinical documentation has been shown to reduce time pressure and improve documentation quality. The extent of time reduction was influenced by the number of voice memos recorded and participants' willingness to adopt the system. This suggests that implementation strategies should account for users' motivation and cooperation to maximize efficiency and cost-effectiveness. Future studies should control for these factors and further investigate the long-term sustainability of generative AI, its potential benefits on healthcare professionals' mental well-being (e.g., stress reduction and burnout prevention), develop standardized metrics for validating AI-generated documentation accuracy and quality, and conduct multi-center collaborative research to ensure generalizability and robustness of findings.
